# The Moderating Effect of Educational Background on the Efficacy of a Computer-Based Brief Intervention Addressing the Full Spectrum of Alcohol Use: Randomized Controlled Trial

**DOI:** 10.2196/33345

**Published:** 2022-06-30

**Authors:** Andreas Staudt, Jennis Freyer-Adam, Christian Meyer, Gallus Bischof, Ulrich John, Sophie Baumann

**Affiliations:** 1 Institute and Policlinic of Occupational and Social Medicine Faculty of Medicine Technische Universität Dresden Dresden Germany; 2 Department of Methods in Community Medicine Institute of Community Medicine University Medicine Greifswald Greifswald Germany; 3 Institute for Medical Psychology University Medicine Greifswald Greifswald Germany; 4 German Centre for Cardiovascular Research Partner Site Greifswald Greifswald Germany; 5 Department of Prevention Research and Social Medicine Institute of Community Medicine University Medicine Greifswald Greifswald Germany; 6 Department of Psychiatry and Psychotherapy University of Lübeck Lübeck Germany

**Keywords:** drinking, brief intervention, screening, school education, public health, prevention

## Abstract

**Background:**

The alcohol-attributable burden of disease is high among socially disadvantaged individuals. Interventional efforts intending to have a public health impact should also address the reduction of social inequalities due to alcohol.

**Objective:**

The aim was to test the moderating role of educational background on the efficacy of a computer-based brief intervention addressing the full spectrum of alcohol use.

**Methods:**

We recruited 1646 adults from the general population aged 18 to 64 years (920 women, 55.9%; mean age 31 years; 574 with less than 12 years of school education, 34.9%) who reported alcohol use in the past year. The participants were randomly assigned a brief alcohol intervention or to assessment only (participation rate, 66.9%, 1646/2463 eligible persons). Recruitment took place in a municipal registry office in one German city. All participants filled out a self-administered, tablet-based survey during the recruitment process and were assessed 3, 6, and 12 months later by study assistants via computer-assisted telephone interviews. The intervention consisted of 3 computer-generated and individualized feedback letters that were sent via mail at baseline, month 3, and month 6. The intervention was based on the transtheoretical model of behavior change and expert system software that generated the feedback letters automatically according to previously defined decision rules. The outcome was self-reported change in number of alcoholic drinks per week over 12 months. The moderator was school education according to highest general educational degree (less than 12 years of education vs 12 years or more). Covariates were sex, age, employment, smoking, and alcohol-related risk level.

**Results:**

Latent growth modeling revealed that the intervention effect after 12 months was moderated by educational background (incidence rate ratio 1.38, 95% CI 1.08-1.76). Individuals with less than 12 years of school education increased their weekly alcohol use to a lesser extent when they received the intervention compared to assessment only (incidence rate ratio 1.30, 95% CI 1.05-1.62; Bayes factor 3.82). No difference was found between groups (incidence rate ratio 0.95, 95% CI 0.84-1.07; Bayes factor 0.30) among those with 12 or more years of school education.

**Conclusions:**

The efficacy of an individualized brief alcohol intervention was moderated by the participants’ educational background. Alcohol users with less than 12 years of school education benefited, whereas those with 12 or more years did not. People with lower levels of education might be more receptive to the behavior change mechanisms used by brief alcohol interventions. The intervention approach may support the reduction of health inequalities in the population at large if individuals with low or medium education can be reached.

**Trial Registration:**

German Clinical Trials Register DRKS00014274; https://www.drks.de/DRKS00014274

## Introduction

Globally, alcohol use is one of the most important risk factors for impaired health [1]. The alcohol-attributable burden of disease has been found to be higher among those with low school education [2,3] or low socioeconomic status [4,5]. Furthermore, for a given amount of alcohol consumed, lower-educated groups have been found to experience disproportionately higher levels of alcohol-related harm, including alcohol-attributable mortality [6,7]. Although the underlying mechanisms of this relationship, commonly referred to as the alcohol harm paradox [8], are not yet fully understood [7,9], reducing social inequalities in alcohol-related harm can be regarded as a major public health concern [10].

Any interventional effort that intends to have a public health impact should address the reduction of social inequalities due to alcohol [11]. In the public health literature, the equity impact has proven to be a useful tool for operationalizing social gradients in intervention effects [12]. The equity impact of interventions can be positive if lower-educated groups are relatively more responsive to the intervention, neutral if the impact is the same for higher- and lower-educated groups, or negative if higher-educated groups are relatively more responsive to the intervention [12]. There is currently no convincing evidence on which types of population-based alcohol interventions, other than tax and price increases or availability restrictions, can reduce inequalities by educational background [13].

Brief alcohol interventions (BAIs) may be a tool to reduce alcohol consumption in populations with low levels of education. The umbrella term “BAI” includes interventions that aim at reducing alcohol-related harm by targeting people’s motivation to change their alcohol use [14]. BAIs have been proven efficacious in reducing alcohol use in primary care populations [15] and have the potential to produce effects in the population at large when disseminated as part of systematic screening [16,17]. Moreover, modern technologies enable the provision of computer-based BAIs to large numbers of recipients at low cost [18]. Although it is known that people with low education are less likely to take up an offered health behavior intervention [19,20], research on the moderating role of education in behavior change intervention effects is scarce [21]. Thus, the equity impact of BAIs warrants further study.

Promising but model-dependent findings from an individual patient data meta-analysis revealed that heavy drinkers with low education who received internet-based interventions had stronger reductions in alcohol consumption compared to more highly educated heavy drinkers [22]. Educational attainment has been found to moderate the strength of the relationship between health cognitions and health behavior [23]. Since health cognitions are a central target of the behavior change techniques in BAIs [24], the way people respond to BAIs might depend on their educational background.

Therefore, the aim of the present study was to shed light on the moderating role of educational background on the efficacy of a computer-based brief intervention addressing the full spectrum of alcohol use. The target group included all alcohol users, irrespective of their alcohol use severity. The rationale of the intervention was based on findings that alcohol consumption has linear dose-response relationships with cancer [25,26] and cardiovascular disease [27]. Thus, motivating a large group of people to maintain or reduce their alcohol use at low levels may produce beneficial public health effects. The intervention was evaluated in a randomized controlled trial using a general population sample [28]. An assessment-only control group was chosen as comparator because repeated assessments, which were necessary for the intervention, may already reduce alcohol consumption [29,30]. To be able to attribute potential effects to the intervention itself, research participation effects [31] had to be controlled for. Although there was no clear evidence for 12-month efficacy [32], intervention effects may vary by educational background.

## Methods

### Ethics Approval

The study was approved by the ethics committee of the University Medicine Greifswald, Germany (protocol number BB 147/15).

### Trial Description

This paper reports outcome data from the 2-armed, parallel-group randomized controlled trial “testing a proactive expert system intervention to prevent and to quit at-risk alcohol use” (PRINT). The study was prospectively registered at the German Clinical Trials Register (DRKS00014274; date of registration March 12, 2018). The corresponding study protocol [28], data on reach and retention [33], and primary outcome analyses [32] can be found elsewhere.

### Participants and Procedure

Between April and June 2018, trial participants were proactively recruited in the waiting area of the municipal registry office in Greifswald, Mecklenburg-West Pomerania, Germany. The registry office is the public authority in charge of registration, passports, and vehicle administration issues. During opening hours, study assistants approached all persons appearing in the waiting area. Those between the ages of 18 and 64 were invited to take part in a tablet-based, self-administered survey on health behaviors. Persons who were already approached during an earlier visit, were cognitively or physically incapable, had insufficient language or reading skills, or were employed at the conducting research institute were excluded.

The survey served as eligibility screening. Individuals who reported alcohol use in the past 12 months were invited to participate in the PRINT trial. Persons without a permanent address or telephone number were excluded. The study assistants informed the eligible individuals about the purpose, procedure, and data handling of the PRINT trial. All participants who gave their written informed consent were randomized to the intervention or assessment-only groups by the tablet computers, using simple randomization (with a 1:1 group allocation ratio) based on a random-number table and the individuals as units of randomization. The allocation sequence was concealed to the study assistants who carried out the recruitment.

The study assistants conducted computer-assisted telephone interviews after 3, 6, and 12 months. After 10 unsuccessful contact attempts, the participants received a questionnaire by email or postal mail, followed by up to 2 written reminders. Participants randomly assigned to the intervention group received computer-generated, individualized feedback letters by postal mail at baseline, month 3, and month 6. All participants received 2 vouchers worth €5 (US $5.34) each as compensation for their participation. One voucher was given out immediately after recruitment in the registry office and the other was sent via postal mail prior to the 12-month follow-up assessment. Participants remained blinded to their individual group assignment until they received the BAI or did not. The study assistants responsible for recruitment, telephone interviews, and management of participant data were blinded to the participants’ group allocation.

### Intervention and Control Groups

The intervention consisted of up to 3 individualized feedback letters (at baseline, month 3, and month 6) based on the transtheoretical model of behavior change [34]. The intervention is described in more detail elsewhere [32]. The letters were generated automatically by expert system software [35], printed, and sent via postal mail. Feedback elements were chosen according to previously defined decision rules based on a participant’s demographic and alcohol-related characteristics. The intervention was designed to address the full spectrum of alcohol use, from low-risk drinkers to participants with probable alcohol use disorder (AUD).

The feedback letters were tailored to the participants’ current alcohol use risk level according to their scores on the Alcohol Use Disorders Identification Test (AUDIT) [36] and its consumption questions, AUDIT-Consumption (AUDIT-C) [37]. All feedback letters included information on recommendations for low-risk alcohol use, with the addendum that “low risk” does not equal “no risk,” as well as written and graphical feedback on the amount of alcohol consumed in comparison to the individual norm group (ie, personalized normative feedback). Participants classified as at-risk drinkers received normative feedback on their motivational stage of change (precontemplation, contemplation, preparation, or action), decisional balance (perceived advantages and disadvantages of reducing alcohol use), self-efficacy, and processes of change [34]. Participants with AUD according to screening (AUDIT score ≥20) received slightly modified feedback that focused on the motivation to utilize professional treatment. Information on local alcohol treatment services was provided. Instead of the feedback given to the at-risk drinkers on the potential risk associated with their individual level of drinking, participants with probable AUD received feedback on symptoms they had already experienced according to the AUDIT. Feedback letters at months 3 and 6 included ipsative feedback delineating the individual development since baseline regarding actual behavior change and changes in motivational measures. The expert system used data gathered in the assessments to generate the feedback letters. Therefore, participating in the respective assessment was required to receive the intervention at that point in time.

The control group received assessment only; in other words, they answered the same tablet-based, self-administered baseline survey (Multimedia Appendix 1 and Multimedia Appendix 2) and computer-assisted telephone interviews at months 3, 6, and 12 as the intervention group.

### Measures

#### Outcome

Change in the number of drinks per week from baseline to month 12 was the primary outcome. This measure was based on self-reported frequency (answering the question “How often did you have a drink containing alcohol in the past 30 days?”) and quantity of alcohol use (answering the question “How many drinks did you have on a typical day when you were drinking?”). The definition of a standard alcoholic drink (0.25 L to 0.3 L beer, 0.1 L to 0.15 L wine or sparkling wine, or 4 cL spirits) was displayed on the tablet screen or read aloud by a study assistant during the interviews. To estimate the average number of drinks per week, frequency was multiplied by typical quantity, divided by 4.25 (ie, the average number of weeks in a month) and rounded down to the nearest integer.

#### Moderator

Participants were asked to indicate their highest general educational degree at baseline. The response options were presented as an exhaustive list of possible school-leaving qualifications in Germany and equivalent foreign degrees, if applicable. The information provided was condensed into a categorical measure of educational background (low: 9 or less years of school education, medium: 10 to 11 years of school education, high: 12 or more years of school education). Due to the unequal distribution of educational background within the sample, the 2 former groups were combined to conduct the moderation analysis with sufficient statistical power. Thus, a binary indicator of educational background (less than 12 years vs 12 or more years of school education) was used. An additional moderation analysis with the 3-category indicator of educational background is reported as a sensitivity analysis in Multimedia Appendix 3.

#### Covariates

Covariates were sex, age, employment status, smoking, and alcohol-related risk level. Participants were asked if they were female or male. Employment status encompassed full-time employment, part-time employment, being a student, unemployment, and other (being retired, a homemaker, or similar). Participants were asked to characterize their own smoking behavior (never, former, occasional, or daily smoking) and occasional and daily smokers were followed up with questions about typical frequency (“How many days per month do you smoke?”) and quantity (“How many cigarettes or comparable tobacco products do you currently smoke on a day when you smoke?”) of smoking. The average number of cigarettes consumed per day was derived as an indicator of smoking. Nonsmokers received a value of zero on that measure. Alcohol-related risk level (low-risk and at-risk) was measured via the AUDIT-C sum score, with sex-specific cut-off values (≥4 for women and ≥5 for men) indicating at-risk alcohol use [38].

### Sample Size Calculation

We hypothesized that there would be a 15% difference between the intervention group (8.5 drinks per week) and control group (10 drinks per week) at the 12-month follow-up. Calculations revealed that if the primary outcome followed a negative binomial distribution with a dispersion parameter of 1.0, 80% power, and 5% significance level, 659 participants per group would be required. With an expected dropout rate of 20%, a total sample size of N=1648 was planned.

### Statistical Analysis

Data were analyzed using latent growth curve modeling (LGM) in Mplus version 7. LGM is designed to analyze interindividual differences in intraindividual change over time. LGM is flexible in handling missing and nonnormally distributed data, as well as complex nonlinear growth trajectories [39]. Growth models were calculated with a full-information maximum likelihood estimator with robust standard errors using all available data (ie, including all baseline participants) assuming that data were missing at random. Thus, all analyses followed an intention-to-treat principle. Due to the positive skewness of the outcome, negative binomial models were calculated. Latent growth factors represented the change in number of alcoholic drinks per week. Rescaled likelihood ratio tests indicated that the model benefited from including higher-order functions (quadratic and cubic), allowing for nonlinear growth over time. Growth factor variances were estimated freely (except for the cubic growth factor). All models were adjusted by time-invariant covariates (sex, age, employment status, and smoking at baseline) and time-variant covariates (alcohol-related risk level at baseline and months 3, 6, and 12).

Study group, educational background, and their interaction were regressed on the growth factors to test if participants with less than 12 years versus 12 or more years of school education showed differential intervention effects. Differences between the intervention and control groups, as well as the interaction effect with educational background, were given as incidence rate ratios (IRRs) with the 95% CI. Additionally, the Bayes factor (BF) was calculated to estimate the sensitivity of the evidence for intervention effects after 12 months among the 2 subgroups [40]. Using the online Dienes calculator [41], the population value was assumed to follow a half-normal distribution for an expected intervention effect of 15%. BF values lower than 0.33 indicated evidence for lack of an effect, values above 3 evidence for the presence of an effect, and values in between indicated data insensitivity [42].

## Results

### Sample Characteristics

**Figure 1:**
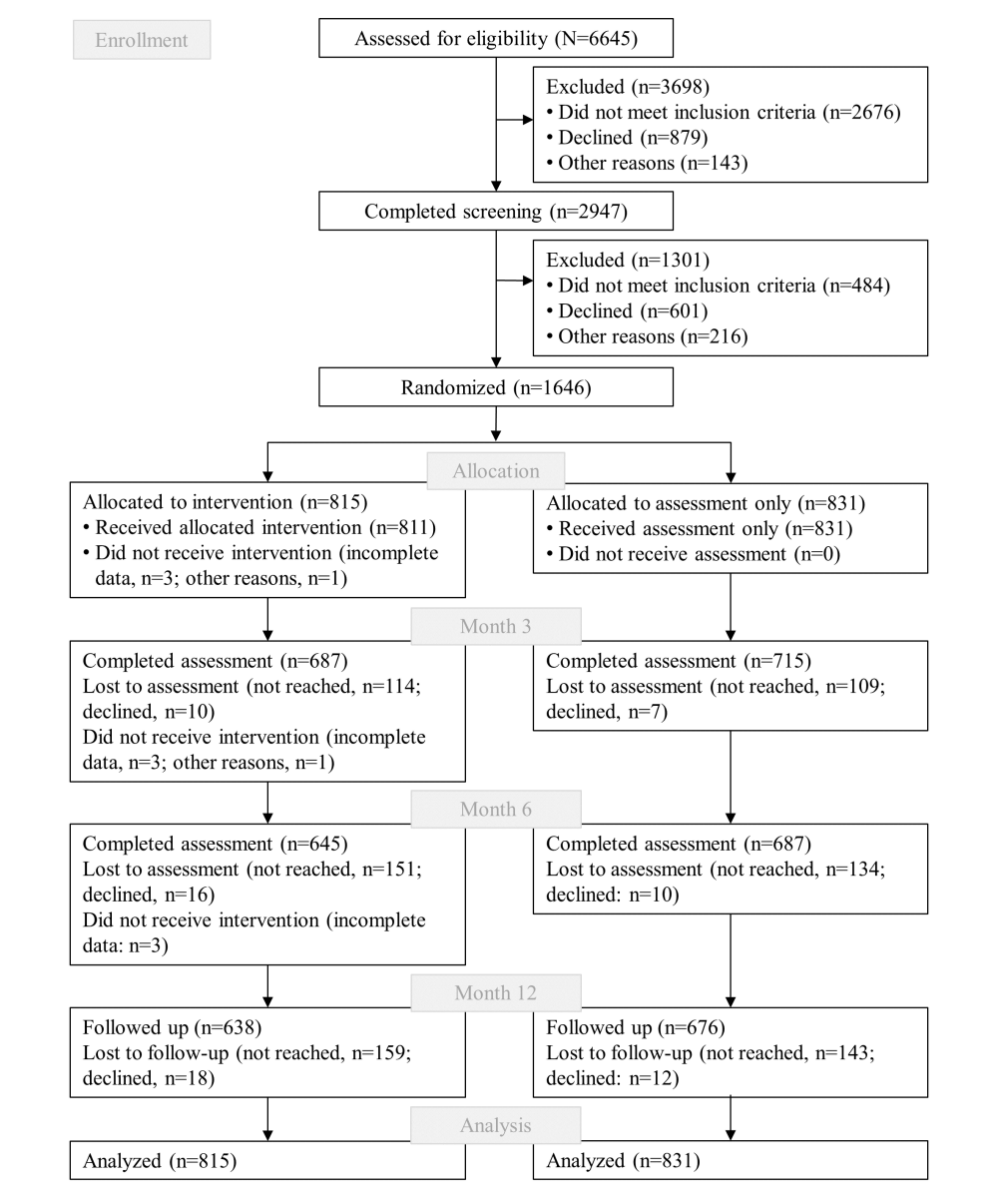
Flow of participants through the trial.

In total, 6645 registry office clients appeared in the waiting area during our recruitment period (Figure 1). Of 3969 clients meeting the inclusion criteria, 2947 (74.3%) completed the PRINT eligibility screening for alcohol use in the previous 12 months. Of 2462 eligible clients, 1646 (66.9%) consented to participate in the trial. Of those 1646 participants, 1406 (85.4%) and 1335 (81.1%) participated in the assessments after 3 and 6 months, respectively. For the 12-month follow-up assessment, 1314 of 1646 (79.8%) participants were reached. The sample (920 women of 1646 participants, 55.9%) had a mean age of 31.0 (SD 10.8) years. Regarding educational background, 574 of 1646 participants (34.9%) had less than 12 years of school education (Table 1).

**Table 1:** Baseline study sample characteristics.

Characteristics		Total sample, N=1646	Less than 12 years of school education, n=574	Twelve or more years of school education, n=1072
Women, n (%)		920 (55.9)	300 (52.3)	620 (57.8)
Age, mean (SD) years		31.0 (10.8)	35.0 (12)	28.9 (9.5)
**School education, n (%)**				
	≤9 years	101 (6.1)	101 (17.6)	N/A^a^
	10 to 11 years	473 (28.7)	473 (82.4)	N/A
	≥12 years	1072 (65.1)	N/A	1072 (100)
**Employment status, n (%)**				
	Employed full-time	689 (41.9)	333 (58)	356 (33.2)
	Employed part-time	358 (21.7)	97 (16.9)	261 (24.4)
	Student	444 (27)	34 (5.9)	410 (38.2)
	Unemployed	53 (3.2)	41 (7.1)	12 (1.1)
	Other	102 (6.2)	69 (12)	33 (3.1)
Cigarettes per day, mean (SD)		3.0 (6.2)	6.0 (8.2)	1.4 (4)
**Alcohol risk level, n (%)**				
	Low-risk alcohol use	1085 (65.9)	423 (73.7)	662 (61.8)
	At-risk alcohol use	561 (34.1)	151 (26.3)	410 (38.2)
Drinks per week, mean (SD)		2.2 (3.9)	1.8 (4.1)	2.4 (3.9)
**Study group, n (%)**				
	Intervention group	815 (49.5)	300 (52.3)	515 (48)
	Control group	831 (50.5)	274 (47.7)	557 (52)

^a^N/A: not applicable.

### Moderation Analysis

**Figure 2:**
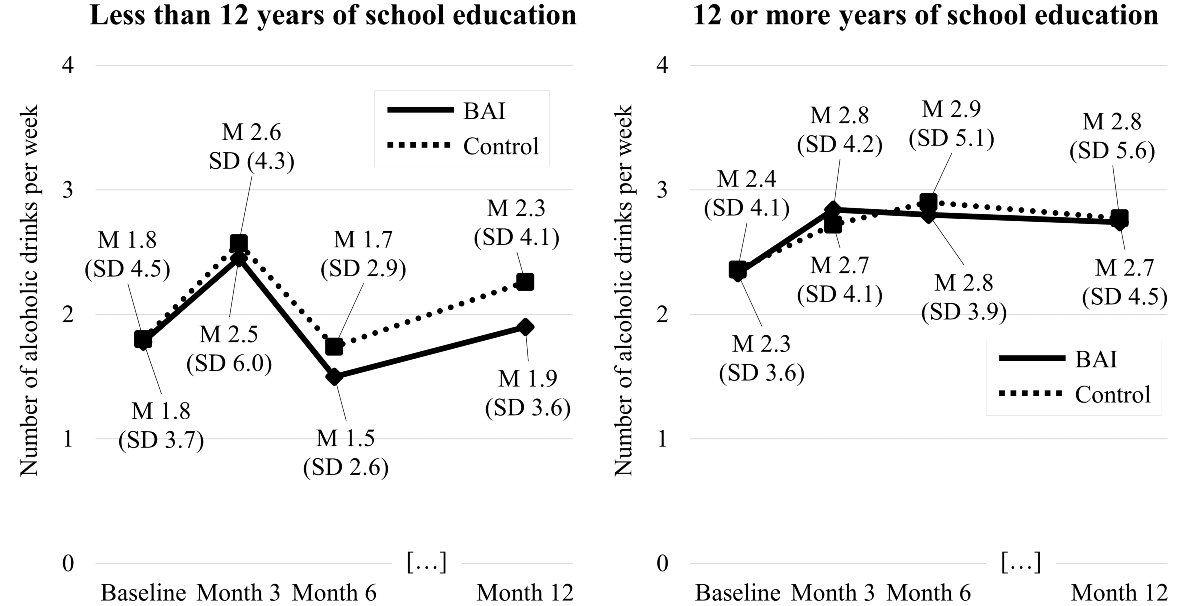
Observed change in number of drinks per week from baseline to month 12 in participants with less than 12 years and 12 or more years of school education. A: less than 12 years of school education; B: 12 or more years of school education; M: mean; BAI: brief alcohol intervention.

Participants with 12 or more years of school education who received the BAI increased their weekly alcohol use from 2.3 (SD 3.6) alcoholic standard drinks at baseline to 2.7 (SD 4.5) drinks at month 12 (Figure 2). BAI group participants with less than 12 years of school education reported 1.8 (SD 3.7) drinks at baseline and 1.9 (SD 3.6) drinks at month 12. Control group participants with 12 or more years of school education increased their weekly alcohol use from 2.4 (SD 4.1) drinks at baseline to 2.8 (SD 5.6) drinks at month 12. An increase was also observed in control group participants with less than 12 years of school education, who reported an average of 1.8 (SD 4.5) drinks at baseline and 2.3 (SD 4.1) drinks at month 12.

**Figure 3:**
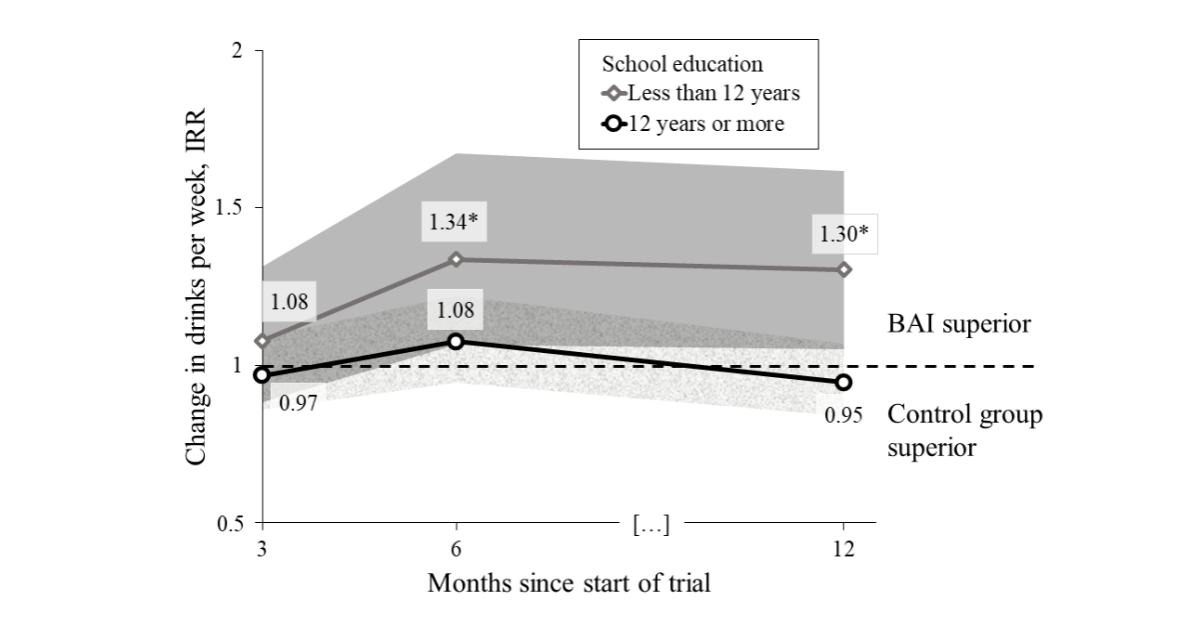
Intervention effect (compared to assessment only) for participants with less than 12 years and 12 or more years of school education. BAI: brief alcohol intervention; IRR: incidence rate ratio.

There was an intervention effect after 12 months in participants with less than 12 years of school education (IRR 1.30, 95% CI 1.05-1.62; BF [0, 0.14] 3.82), but not among participants with 12 or more years of school education (IRR 0.95, 95% CI 0.84-1.07; BF [0, 0.14] 0.30). Figure 3 illustrates the intervention effects as IRRs over time, with the shaded areas indicating 95% CI. Participants with less than 12 years of school education were significantly more likely to benefit from the intervention after 12 months compared to participants with 12 or more years of school education (IRR 1.38, 95% CI 1.08-1.76; *P*=.03) (Table 2). There was no significant interaction effect during the active intervention phase, either at month 3 (IRR 1.24, 95% CI 0.96-1.61; *P*=.44) or at month 6 (IRR 1.11, 95% CI 0.88-1.40; *P*=.17).

The results of an additional moderation analysis with a 3-category indicator of educational background (low: 9 or less years of school education, medium: 10 to 11 years of school education, high: 12 or more years of school education) can be found in Multimedia Appendix 3.

**Table 2:** Intervention effects over 12 months were moderated by educational background^a^.

Time points			Difference between intervention and control group, incidence rate ratio (95% CI)			
			Less than 12 years of school education		Twelve or more years of school education	Interaction effect
**Active intervention phase**						
	Month 3	1.08 (0.88-1.32)		0.97 (0.86-1.09)		1.11 (0.88-1.40)
	Month 6	1.34 (1.07-1.68)		1.08 (0.95-1.22)		1.24 (0.96-1.61)
Follow-up (month 12)			1.30 (1.05-1.62)		0.95 (0.84-1.07)	1.38 (1.08-1.76)

^a^Latent growth model (N=1646) with higher-order growth factors for negative binomial distributed outcome data. The outcome was net change in number of alcoholic drinks per week. The model was adjusted for sex, age, employment status, smoking, and alcohol-related risk level. Incidence rate ratios with 95% CI are displayed.

## Discussion

### Principal Findings

The efficacy of a computer-based BAI addressing the full spectrum of alcohol use was moderated by educational background. After 12 months, alcohol users from the general population with lower school education benefited from the intervention, whereas those with higher school education did not. These findings allow the presumption that BAIs might be able to support the reduction of social inequalities due to alcohol. The present study showed that an individualized BAI based on expert system software was effective among study participants with lower school education.

### Comparison With Prior Work

To our knowledge, this is the first study to investigate the efficacy of a BAI in general population subgroups with different educational backgrounds. Previous studies focused mainly on other treatment moderators, such as sex, age, and consumption-related variables [43-46], but neglected school education as a potential moderator. Comparable evidence comes from a recent meta-analysis whose findings supported the notion that internet-based interventions may be particularly beneficial for heavy drinkers with a low educational background [22]. In contrast, a technology-based intervention targeting heavy drinking was found to be more effective for highly educated adolescents in Switzerland, compared to less-educated adolescents [47]. Notwithstanding these findings, the interaction of school education and BAI efficacy is not yet well understood [21]. This study contributes to the sparse literature by showing that a BAI based on expert system software is effective among alcohol users with low and medium education.

People with a lower educational background might be more receptive to the behavior change mechanisms included in BAIs. Underestimating one’s alcohol use relative to others (ie, normative misperception) has been found to be more pronounced among less-educated alcohol users [48]. If normative misperceptions precede and encourage alcohol use [49], correcting this fallacy by means of personalized normative feedback might reduce alcohol use over time [50], in particular among individuals who are more prone to believe that others drink more frequently and consume more alcohol than themselves. Personalized normative feedback was a central component of the intervention tested in this study [32]. Individuals with different educational backgrounds might have responded differently to this personalized normative feedback, possibly explaining the interaction between educational background and intervention efficacy. Feedback that compares alcohol use between an an individual and their peer group might have a stronger motivating effect to reduce drinking in people with less than 12 years of school education than in those with 12 or more years of school education. Moreover, less-educated individuals might have to justify their alcohol use more often and be denied autonomy over their alcohol use more often. The BAI was designed to incorporate the spirit of motivational interviewing [51] by being centered on the participants’ own point of view and valuing their motives and attitudes regarding their alcohol use. Feedback was provided in an appreciative manner, such as by pointing out the subjective advantages and disadvantages of the participants’ alcohol use. This experience of appreciation might have been more motivating for less-educated compared to higher-educated individuals.

The findings speak in favor of the view that population-based BAIs might have a positive equity impact. Addressing the alcohol harm paradox is a major public health issue [10]. BAIs might be a piece of the puzzle on the path to reducing social inequality due to alcohol if (a) they are disseminated with a systematic screening approach and (b) they reach a substantial part of the population with low school education. However, it is known that lower-educated individuals are less likely to take up an offered intervention [19,20], as was the case in the PRINT trial [33]. The percentage of participants who received the complete intervention, consisting of all 3 feedback letters, was higher among those with high (413/515, 80%) education than those with low or medium education (202/300, 67%). The latter were also more difficult to reach for the telephone interviews that were needed to deliver the intervention. Therefore, strategies need to be focused on how people with a lower educational background can be reached and retained for alcohol prevention. Settings may be chosen where less-educated individuals can be reached, such as job centers [52] or primary health care clinics [53], and are best combined with a proactive approach [54].

The intervention effect after 12 months was small in magnitude, possibly because the study was not restricted to at-risk alcohol users but targeted the full spectrum of alcohol use. Thus, the initial drinking level was lower than in previous BAI trials [15], resulting in a smaller margin for reduction in alcohol consumption. It must be acknowledged that it remains unclear if BAIs will diminish social inequalities in alcohol-attributable harm by addressing alcohol use per se. Consumption-related factors may not be sufficient to explain the alcohol harm paradox [55]. Rather, a more holistic view is needed, taking into account interactions with other health behaviors [8] and social risk factors such as deprivation [56].

### Limitations

This study has several strengths and limitations. The findings add to the sparse evidence on educational background as a moderator of BAI efficacy. High participation and retention rates in a general population sample ensured external validity. The intervention approach was novel, as it addressed the full spectrum of alcohol use, not only in at-risk drinkers. The limitations were 4-fold. First, selection bias was likely, since baseline factors such as alcohol-related risk level are associated with trial participation [33]. Second, all data were completely self-reported. Third, the main outcome was measured with a quantity-frequency approach that might have underestimated the true amount of alcohol consumed [57]. Fourth, this was a secondary data analysis. The PRINT trial was not designed or powered to scrutinize how the intervention worked in subgroups with different educational backgrounds. As lower-educated people were underrepresented in our sample, comparing more than 2 subgroups resulted in a loss of statistical power, wider confidence intervals, and data insensitivity for differential efficacy (additional moderation analysis is shown in Multimedia Appendix 3).

### Conclusions

The present study provided insight into the role of educational background in BAI efficacy in the general population. Future research might investigate the circumstances under which the expected positive equity impact of BAIs can be maximized. The intervention approach might be able to reduce health inequalities due to alcohol in the population at large if people with low or medium education can be reached.

## Appendix

Multimedia Appendix 1 German questionnaire.

Multimedia Appendix 2 English questionnaire.

Multimedia Appendix 3 Additional moderation analysis.

Multimedia Appendix 4 CONSORT-eHEALTH checklist (V 1.6.1).

## Abbreviations

AUD: alcohol use disorder
AUDIT: Alcohol Use Disorders Identification Test
AUDIT-C: Alcohol Use Disorders Identification Test-Consumption
BAI: brief alcohol intervention
IRR: incidence rate ratio
LGM: latent growth curve modeling
PRINT: proactive expert system intervention to prevent and to quit at-risk alcohol use
