# Supplementary material for: The Moderating Effect of Educational Background on the Efficacy of a Computer-Based Brief Intervention Addressing the Full Spectrum of Alcohol Use: Randomized Controlled Trial
Source: JMIR Public Health Surveill. 2022 Jun 30;8(6):e33345. doi: 10.2196/33345 (PMC9284353; doi:10.2196/33345)
Supplement: Multimedia Appendix 1 [file publichealth_v8i6e33345_app1.pdf]

ID

Forschungsprojekt

print

Fragebogen der PRINT-Studie

This is a Multimedia Appendix to a full manuscript published in JMIR Public Health Surveill. For full copyright and citation information see http://dx.doi.org/10.2196/jmir.33345

Datum: ___ . ___ . 2018

1. Sind Sie:

- ☐0 Männlich
- ☐1 Weiblich

2. Wie alt sind Sie?

_____ Jahre

3. Wie würden Sie Ihren Gesundheitszustand im Allgemeinen beschreiben?

- ☐1 Ausgezeichnet
- ☐2 Sehr gut
- ☐3 Gut
- ☐4 Weniger gut
- ☐5 Schlecht

4. Geben Sie bitte anhand der Skala an, wie häufig Sie im letzten Monat die folgenden Stimmungen hatten.[1]

Wie häufig im letzten Monat ….	nie 1	selten 2	gelegent-lich 3	oft 4	immer 5
… waren Sie sehr nervös?	☐	☐	☐	☐	☐
… haben Sie sich ruhig und gelassen gefühlt?	☐	☐	☐	☐	☐
… haben Sie sich niedergeschlagen und traurig gefühlt?	☐	☐	☐	☐	☐
… waren Sie sehr glücklich?	☐	☐	☐	☐	☐
… haben Sie sich so niedergeschlagen gefühlt, dass Sie nichts aufheitern konnte?	☐	☐	☐	☐	☐

5. Sind Sie zurzeit Raucher?

- ☐0 Nein, ich habe nie geraucht ⇨ *Weiter mit Frage 7*
- ☐1 Nein, ich rauche nicht mehr ⇨ *Weiter mit Frage 7*
- ☐2 Ja, ich rauche <u>täglich</u> ⇨ *Weiter mit Frage 6*
- ☐3 Ja, ich rauche <u>gelegentlich</u> ⇨ **An wie vielen Tagen im Monat rauchen Sie?**
  an _____Tagen ⇨ *Weiter mit Frage 6*

6. Wie viele Zigaretten/ Zigarillos/ Pfeife/ Zigarren rauchen Sie derzeit üblicherweise an einem Tag, an dem Sie rauchen?

_____ Stück

7. Jetzt denken Sie bitte an Ihren Alkoholkonsum in der vergangenen Woche. Wie viele alkoholische Getränke haben Sie an jedem Tag in der vergangenen Woche, d.h. an den letzten 7 Tagen, getrunken?[2]

Ein alkoholisches Getränk entspricht z.B. 1 Bier 0,25-0,3l oder 1 Wein/Sekt 0,1-0,15l oder 1 doppelter Schnaps/Likör 4cl. Rechnen Sie große Getränke in kleine um, z.B. 1 Flasche/Glas Bier à 0,5l sind 2 Getränke. Bei Nichtzutreffen tragen Sie eine Null ein.

Tragen Sie dazu hier das heutige Datum ein: ___ . ___ . 2018 und beziehen Ihre Angabe auf die zurückliegenden 7 Tage.

Tag	Anzahl
gestern (Datum: ___ . ___ . 2018)	
vorgestern (Datum: ___ . ___ . 2018)	
vor 3 Tagen (Datum: ___ . ___ . 2018)	
vor 4 Tagen (Datum: ___ . ___ . 2018)	
vor 5 Tagen (Datum: ___ . ___ . 2018)	
vor 6 Tagen (Datum: ___ . ___ . 2018)	
vor 7 Tagen (Datum: ___ . ___ . 2018)	

8. Nun folgen einige weitere Fragen zum Alkoholtrinken. Alle Teilnehmer/innen dieser Umfrage erhalten die gleichen Fragen. Wir bitten Sie deshalb, auch diejenigen Fragen zu beantworten, bei denen Sie das Gefühl haben, dass sie überhaupt nicht auf Ihre persönliche Situation zutreffen.

Wie oft nehmen Sie in alkoholisches Getränk zu sich?[3]

☐0 Niemals ⇨ **Auch dann nicht, wenn Sie z.B. den Wein zum Essen, das Bier bei einer Sportveranstaltung oder den Sekt bei Feierlichkeiten mitzählen?**

- ☐1 Ja, auch dann nicht ⇨ *Weiter mit Frage 18*
- ☐0 Nein, da habe ich etwas getrunken ⇨ *Frage 8 wiederholen*

☐1 1mal im Monat oder seltener
☐2 2 bis 4mal im Monat
☐3 2 bis 3mal pro Woche
☐4 4mal oder häufiger in der Woche

9. **Wenn Sie alkoholische Getränke zu sich nehmen, wie viel trinken Sie dann typischerweise an einem Tag?**[3] Ein alkoholisches Getränk entspricht z.B. 1 Bier 0,25-0,3l oder 1 Wein/Sekt 0,1-0,15l oder 1 doppelter Schnaps/ Likör 4cl.

- ☐0 1 - 2
- ☐1 3 - 4
- ☐2 5 - 6
- ☐3 7 - 9
- ☐4 10 oder mehr

10. **Wenn Sie eine Frau sind: Wie oft trinken Sie 4 oder mehr alkoholische Getränke zu einer Gelegenheit?**[3]

Wenn Sie ein Mann sind: Wie oft trinken Sie 5 oder mehr alkoholische Getränke zu einer Gelegenheit?[3]

- ☐0 Niemals
- ☐1 Seltener als 1mal im Monat
- ☐2 1mal im Monat
- ☐3 1mal pro Woche
- ☐4 Täglich oder fast täglich

11. **Wie oft haben Sie in den letzten 12 Monaten erlebt, dass Sie nicht mehr mit dem Trinken aufhören konnten, nachdem Sie einmal begonnen hatten?**[3]

- ☐0 Niemals
- ☐1 Seltener als 1mal im Monat
- ☐2 1mal im Monat
- ☐3 1mal pro Woche
- ☐4 Täglich oder fast täglich

12. **Wie oft passierte es in den letzten 12 Monaten, dass Sie wegen des Trinkens Erwartungen, die man an Sie in der Familie, im Freundeskreis und im Berufsleben hat, nicht mehr erfüllen konnten?**[3]

- ☐0 Niemals
- ☐1 Seltener als 1mal im Monat
- ☐2 1mal im Monat
- ☐3 1mal pro Woche
- ☐4 Täglich oder fast täglich

13. **Wie oft brauchten Sie in den letzten 12 Monaten am Morgen ein erstes Glas, um sich nach einem Abend mit viel Alkoholgenuss wieder fit zu fühlen?**[3]

- ☐0 Niemals
- ☐1 Seltener als 1mal im Monat
- ☐2 1mal im Monat
- ☐3 1mal pro Woche
- ☐4 Täglich oder fast täglich

14. Wie oft hatten Sie in den letzten 12 Monaten wegen Ihrer Trinkgewohnheiten Schuldgefühle oder Gewissensbisse?[3]

- ☐0 Niemals
- ☐1 Seltener als 1mal im Monat
- ☐2 1mal im Monat
- ☐3 1mal pro Woche
- ☐4 Täglich oder fast täglich

15. Wie oft haben Sie sich während der letzten 12 Monate nicht mehr an den vorangegangenen Abend erinnern können, weil Sie getrunken hatten?[3]

- ☐0 Niemals
- ☐1 Seltener als 1mal im Monat
- ☐2 1mal im Monat
- ☐3 1mal pro Woche
- ☐4 Täglich oder fast täglich

16. Haben Sie sich oder eine andere Person unter Alkoholeinfluss schon mal verletzt?[3]

- ☐0 Nein
- ☐2 Ja, aber nicht in den letzten 12 Monaten
- ☐4 Ja, in den letzten 12 Monaten

17. Hat ein Verwandter, Freund oder auch ein Arzt schon einmal Bedenken wegen Ihres Trinkverhaltens geäußert?[3]

- ☐0 Nein
- ☐2 Ja, aber nicht in den letzten 12 Monaten
- ☐4 Ja, in den letzten 12 Monaten

18. Jetzt rufen Sie sich bitte Ihren Alkoholkonsum <u>der letzten 30 Tage</u> in Erinnerung

Wie oft nahmen Sie in den letzten 30 Tagen ein alkoholisches Getränk zu sich?

- ☐0 Niemals
- ☐1 1mal im Monat
- ☐2 2 bis 4mal im Monat
- ☐3 2 bis 3mal pro Woche
- ☐4 Täglich oder fast täglich

19. Wenn Sie in den letzten 30 Tagen getrunken haben, wie viele Getränke tranken Sie dann <u>typischerweise</u> an solch <u>einem Tag</u>? Ein alkoholisches Getränk entspricht z.B. 1 Bier 0,25-0,3l oder 1 Wein/Sekt 0,1-0,15l oder 1 doppelter Schnaps/Likör 4cl.

____ Getränke

20. Haben Sie in den letzten 3 Monaten ernsthaft versucht, dauerhaft weniger Alkohol zu trinken oder ganz aufzuhören?

☐1 Ja ⇨ **Hat der letzten Versuch bis heute angehalten?**

- ☐1 Ja ⇨ *Weiter mit Frage 23*
- ☐0 Nein ⇨ *Weiter mit Frage 21*

☐0 Nein ⇨ *Weiter mit Frage 22*

21. Welche Aussage trifft derzeit am besten auf Sie zu?

☐1 Ich habe nicht vor, dauerhaft weniger Alkohol zu trinken ⇨ *Weiter mit Frage 22*
☐2 Ich denke darüber nach, dauerhaft weniger Alkohol zu trinken ⇨ *Frage 23*
☐3 Ich plane fest, dauerhaft weniger Alkohol zu trinken ⇨ *Frage 23*

22. Denken Sie, dass Sie mehr trinken, als Sie sollten?

☐0 Nein
☐1 Ja

23. Sind Sie zurzeit…

☐1 ledig ⇨ *Weiter mit Frage 24*
☐2 verheiratet und lebe mit Ehepartner/in zusammen ⇨ *Weiter mit Frage 26*
☐3 verheiratet und lebe von Ehepartner/in getrennt ⇨ *Weiter mit Frage 26*
☐4 geschieden ⇨ *Weiter mit Frage 24*
☐5 verwitwet ⇨ *Weiter mit Frage 24*

24. Leben Sie derzeit in einer festen Partnerschaft?

☐0 Nein ⇨ *Weiter mit Frage 26*
☐1 Ja ⇨ *Weiter mit Frage 25*

25. Leben Sie mit Ihrem/Ihrer Partner/in zusammen?

☐0 Nein
☐1 Ja

26. Welchen höchsten allgemeinbildenden Schulabschluss haben Sie erreicht?

- ☐0 Keinen, ich gehe auch nicht mehr zur Schule ⇨ *Weiter mit Frage 28*
- ☐1 Volks-/ Hauptschule/ POS 8./9. Klasse ⇨ *Weiter mit Frage 28*
- ☐2 Realschule/ POS ⇨ *Weiter mit Frage 28*
- ☐3 Fachhochschulreife ⇨ *Weiter mit Frage 28*
- ☐4 Abitur/ EOS, Hochschulreife ⇨ *Weiter mit Frage 28*
- ☐5 Einen anderen Schulabschluss ⇨ *Weiter mit Frage 27*
- ☐6 Keinen, ich gehe noch zur Schule ⇨ *Weiter mit Frage 28*

27. Welchen anderen Schulabschluss haben Sie?

- ☐1 Erweiterter Hauptschulabschluss
- ☐2 Erweiterter Realschulabschluss
- ☐3 Förderschule/ Sonderschule/ Hilfsschule
- ☐4 Ausländischer Abschluss vergleichbar mit Hauptschule
- ☐5 Ausländischer Abschluss vergleichbar mit Realschule
- ☐6 Ausländerischer Abschluss vergleichbar mit Abitur
- ☐7 Sonstiges: ______________________

28. Welches ist Ihr höchster beruflicher Abschluss?

- ☐0 Noch in Ausbildung (z.B. Student/in, Auszubildende/r)
- ☐1 Kein Abschluss
- ☐2 Abschlossene Lehre (z.B. Facharbeiter)
- ☐3 Berufsfach-/ Handelsschule
- ☐4 Meister/ Techniker/ Fachschule
- ☐5 Fachhochschulabschluss
- ☐6 Universitätsabschluss

29. Sind Sie derzeit an einer Hochschule (Universität oder Fachhochschule) als Student/in eingeschrieben?

- ☐0 Nein
- ☐1 Ja

30. Sind Sie derzeit erwerbstätig?

- ☐0 Nein ⇨ *Weiter mit Frage 32*
- ☐1 Ja ⇨ *Weiter mit Frage 31*

31. Sind Sie…

- ☐1 …in Vollzeit erwerbstätig (ab 35h/Woche)
- ☐2 …in Teilzeit erwerbstätig (15-34h/Woche)
- ☐3 …geringfügig erwerbstätig
- ☐4 …in beruflicher Ausbildung/ Lehre
- ☐5 …in Umschulung
- ☐6 …im Wehr-/ Bundesfreiwilligendienst/ FSJ
- ☐7 …in Mutterschutz/ Elternzeit

32. Sind Sie…

- ☐1 Schüler/in einer allgemeinbildenden Schule
- ☐2 Student/in
- ☐3 Rentner/in, Pensionär/in, im Vorruhestand
- ☐4 arbeitslos
- ☐5 Hausfrau/ Hausmann
- ☐6 in Mutterschutz/ Elternzeit
- ☐7 Sonstiges: ______________________________

References

1. Rumpf H-J, Meyer C, Hypke U, John U. Screening for mental health: validity of the MHI-5 using DSM-IV Axis I psychiatric disorders as gold standard. Psychiatry Research 2001; 105(3): 243-253. doi: 10.1016/S0165-1781(01)00329-8
2. Sobell LC, Sobell M. Timeline follow-back: a technique for assessing self-reported alcohol consumption. In: Litten RZ, Allen JP (Eds.), Measuring Alcohol Consumption: Psychosocial and Biochemical Methods. 1992; 41-72. Humana Press, Totowa, NJ.
3. Saunders JB, Aasland OG, Babor TF, La Fuente JR de, Grant M. Development of the Alcohol Use Disorders Identification Test (AUDIT): WHO Collaborative Project on Early Detection of Persons with Harmful Alcohol Consumption. Addiction 1993; 88: 791-804. doi:10.1111/j.1360-0443.1993.tb02093.x
