# Supplementary material for: The Moderating Effect of Educational Background on the Efficacy of a Computer-Based Brief Intervention Addressing the Full Spectrum of Alcohol Use: Randomized Controlled Trial
Source: JMIR Public Health Surveill. 2022 Jun 30;8(6):e33345. doi: 10.2196/33345 (PMC9284353; doi:10.2196/33345)
Supplement: Multimedia Appendix 2 [file publichealth_v8i6e33345_app2.pdf]

ID

Research project

print

Questionnaire of the PRINT study

[This questionnaire is a translation of the original questionnaire and was not actually used as the trial was completely conducted in German.]

This is a Multimedia Appendix to a full manuscript published in JMIR Public Health Surveill. For full copyright and citation information see http://dx.doi.org/10.2196/jmir.33345

Date: ___ . ___ . 2018

1. Are you:

- ☐0 Male
- ☐1 Female

2. How old are you?

____ years

3. How would you describe your state of health in general?

- ☐1 Excellent
- ☐2 Very good
- ☐3 Good
- ☐4 Not so good
- ☐5 Bad

4. Using the scale below, please indicate how often you had the following moods in the last month.[1]

How much time of the last month have you ….	None of the time 1	2	3	4	All of the time 5
… been a very nervous person?	☐	☐	☐	☐	☐
… felt calm and peaceful?	☐	☐	☐	☐	☐
… felt downhearted and blue?	☐	☐	☐	☐	☐
… been a happy person?	☐	☐	☐	☐	☐
… felt so down in the dumps that nothing could cheer you up?	☐	☐	☐	☐	☐

5. Do you smoke?

- ☐0 No, I have never smoked ⇨ *Continue with question 7*
- ☐1 No, I do not smoke anymore ⇨ *Continue with question 7*
- ☐2 Yes, I smoke daily ⇨ *Continue with question 6*
- ☐3 Yes, I smoke occasionally ⇨ **How many days a month do you smoke?**

  ____days ⇨ *Continue with question 6*

6. How many cigarettes/ cigarillos/ pipe/ cigars do you currently usually smoke in a day that you smoke?

____ units

7. Now please think about your alcohol consumption in the past week. How many alcoholic drinks did you have on each day in the past week, i.e. in the last 7 days?[2]

One alcoholic drink corresponds to e.g. 1 beer 0.25-0.3l or 1 wine/champagne 0.1-0.15l or 1 liqueur 4cl. Convert large drinks into small ones, e.g. 1 bottle/glass of beer à 0.5l is 2 drinks. If not applicable, enter zero.

Enter today's date here: ___ . ___ . 2018 and relate your answers to the past 7 days

Day	Drinks
yesterday (Date: ___ . ___ . 2018)	
2 days ago (Date: ___ . ___ . 2018)	
3 days ago (Date: ___ . ___ . 2018)	
4 days ago (Date: ___ . ___ . 2018)	
5 days ago (Date: ___ . ___ . 2018)	
6 days ago (Date: ___ . ___ . 2018)	
7 days ago (Date: ___ . ___ . 2018)	

8. All participants receive the same questions. We therefore ask you to also answer those questions that you feel do not apply to your personal situation at all.

How often do you have a drink containing alcohol?[3]

- ☐0 Never ⇨ **Not even if you count, e.g., the wine with the meal, the beer at a sporting event or the champagne at a celebration?**
  - ☐1 Yes, not even then ⇨ *Continue with question 18*
  - ☐0 No, I had a drink then ⇨ *Repeat question 8*
- ☐1 Monthly or less
- ☐2 2-4 times a month
- ☐3 2-3 times a week
- ☐4 4 or more times a week

**9.**	**How many standard drinks do you have on typical day when drinking?**[3] One alcoholic drink corresponds to e.g. 1 beer 0.25-0.3l or 1 wine/champagne 0.1-0.15l or 1 liqueur 4cl.

☐0 1 - 2
☐1 3 - 4
☐2 5 - 6
☐3 7 - 9
☐4 10 or more

**10.**	**If you are a woman: How often do you have <u>4 or more drinks</u> on one occasion?**[3] **If you are no woman: How often do you have <u>5 or more drinks</u> on one occasion?**[3]

☐0 Never
☐1 Less than monthly
☐2 Monthly
☐3 Weekly
☐4 Daily or almost daily

**11.**	**During the past year, how often have you found that you were not able to stop drinking once you had started?**[3]

☐0 Never
☐1 Less than monthly
☐2 Monthly
☐3 Weekly
☐4 Daily or almost daily

**12.**	**During the past year, how often have you failed to do what was normally expected of you because of drinking?**[3]

☐0 Never
☐1 Less than monthly
☐2 Monthly
☐3 Weekly
☐4 Daily or almost daily

**13.**	**During the past year, how often have you needed a drink in the morning to get yourself going after a heavy drinking session?**[3]

☐0 Never
☐1 Less than monthly
☐2 Monthly
☐3 Weekly
☐4 Daily or almost daily

14. During the past year, how often have you had a feeling of guilt or remorse after drinking?[3]

- ☐0 Never
- ☐1 Less than monthly
- ☐2 Monthly
- ☐3 Weekly
- ☐4 Daily or almost daily

15. During the past year, have you been unable to remember what happened the night before because you had been drinking?[3]

- ☐0 Never
- ☐1 Less than monthly
- ☐2 Monthly
- ☐3 Weekly
- ☐4 Daily or almost daily

16. Have you or someone else been injured as a result of your drinking?[3]

- ☐0 No
- ☐2 Yes, but not in the past year
- ☐4 Yes, during the past year

17. Has a relative or friend, doctor or other health worker been concerned about your drinking or suggested you cut down?[3]

- ☐0 No
- ☐2 Yes, but not in the past year
- ☐4 Yes, during the past year

18. Now please recall your alcohol consumption in the past 30 days.

How often did you have a drink containing alcohol in the past 30 days?

- ☐0 Never
- ☐1 Once
- ☐2 2-4 times a month
- ☐3 2-3 times a week
- ☐4 Daily or almost daily

19. In the past 30 days, how many drinks did you have on a typical day when you were drinking? One alcoholic drink corresponds to e.g. 1 beer 0.25-0.3l or 1 wine/champagne 0.1-0.15l or 1 liqueur 4cl.

_____ drinks

20. In the past 3 months, have you seriously attempted to cut down or stop drinking permanently?

☐1 Yes ⇨ **Did the attempt last until today?**

☐1 Yes ⇨ *Continue with question 23*

☐0 Nein ⇨ *Continue with question 21*

☐0 No ⇨ *Continue with question 22*

21. Which statement best applies to you at the moment?

☐1 I do not intend to drink less alcohol ⇨ *Continue with question 22*

☐2 I am thinking about drinking less alcohol ⇨ *Continue with question 23*

☐3 I plan to drink less alcohol ⇨ *Continue with question 23*

22. Do you think you drink more than you should?

☐0 No

☐1 Yes

23. Are you…

☐1 Single ⇨ *Continue with question 24*

☐2 Married and living together with your spouse ⇨ *Continue with question 26*

☐3 Married and living apart ⇨ *Continue with question 26*

☐4 Divorced ⇨ *Continue with question 24*

☐5 Widowed ⇨ *Continue with question 24*

24. Are you currently living in a relationship?

☐0 No ⇨ *Continue with question 26*

☐1 Yes ⇨ *Continue with question 25*

25. Do you live with your partner?

☐0 No

☐1 Yese

26. Which is your highest general educational degree?

- ☐0 None, I don't go to school anymore either ⇨ *Continue with question 28*
- ☐1 Hauptschule, POS (Lower secondary education leaving after class 8 or 9) ⇨ *Continue with question 28*
- ☐2 Realschule, POS (High-school diploma) ⇨ *Continue with question 28*
- ☐3 Fachhochschulreife (University of applied sciences entrance qualification) ⇨ *Continue with question 28*
- ☐4 Abitur (Higher education entrance qualification) ⇨ *Continue with question 28*
- ☐5 Another school-leaving qualification ⇨ *Continue with question 27*
- ☐6 None, I still go to school ⇨ *Continue with question 28*

27. Which other school-leaving qualification do you have?

- ☐1 Erweiterter Hauptschulabschluss (Extended lower secondary degree)
- ☐2 Erweiterter Realschulabschluss (Extended high-school diploma)
- ☐3 School for children with special needs
- ☐4 Foreign degree comparable to lower secondary education
- ☐5 Foreign degree comparable to high-school diploma
- ☐6 Foreign degree comparable to higher education entrance qualification
- ☐7 Other: ________________________

28. Which is your highest professional degree?

- ☐0 Still in training (e.g. university student, apprentice)
- ☐1 No degree
- ☐2 Completed vocational training
- ☐3 Vocational school (technical or commercial college)
- ☐4 Foreman/ technician/ professional school
- ☐5 University of applied sciences degree
- ☐6 University degree

29. Are you currently enrolled as a university student?

- ☐0 No
- ☐1 Yes

30. Are you employed?

- ☐0 No ⇨ *Continue with question 32*
- ☐1 Yes ⇨ *Continue with question 31*

31. Are you…

- ☐1 …full-time employed (35h/week or more)
- ☐2 …part-time employed (15-34h/week)
- ☐3 …marginally employed
- ☐4 …in vocational training
- ☐5 …in occupational retraining
- ☐6 …in military service or federal volunteer service
- ☐7 …in parental leave

32. Are you…

- ☐1 Student in a school of general education
- ☐2 University student
- ☐3 Retiree/ pensioner
- ☐4 Unemployed
- ☐5 Homemaker
- ☐6 in parental leave
- ☐7 Other: ______________________________

References

1. Rumpf H-J, Meyer C, Hypke U, John U. Screening for mental health: validity of the MHI-5 using DSM-IV Axis I psychiatric disorders as gold standard. Psychiatry Research 2001; 105(3): 243-253. doi: 10.1016/S0165-1781(01)00329-8
2. Sobell LC, Sobell M. Timeline follow-back: a technique for assessing self-reported alcohol consumption. In: Litten RZ, Allen JP (Eds.), Measuring Alcohol Consumption: Psychosocial and Biochemical Methods. 1992; 41-72. Humana Press, Totowa, NJ.
3. Saunders JB, Aasland OG, Babor TF, La Fuente JR de, Grant M. Development of the Alcohol Use Disorders Identification Test (AUDIT): WHO Collaborative Project on Early Detection of Persons with Harmful Alcohol Consumption. Addiction 1993; 88: 791-804. doi:10.1111/j.1360-0443.1993.tb02093.x
