# Supplementary material for: The Moderating Effect of Educational Background on the Efficacy of a Computer-Based Brief Intervention Addressing the Full Spectrum of Alcohol Use: Randomized Controlled Trial
Source: JMIR Public Health Surveill. 2022 Jun 30;8(6):e33345. doi: 10.2196/33345 (PMC9284353; doi:10.2196/33345)
Supplement: Multimedia Appendix 3 [file publichealth_v8i6e33345_app3.pdf]

## Additional moderation analysis

This is a Multimedia Appendix to a full manuscript published in JMIR Public Health Surveill. For full copyright and citation information see http://dx.doi.org/10.2196/jmir.33345

## Method

Hereafter, the results of an additional moderation analysis are reported. The analytical strategy was similar to the main analysis with the exception that educational background included three (low: 9 or less years of school education, medium: 10 to 11 years of school education, high: 12 or more years of school education) instead of two categories.

## Results

Of 1,646 participants, 101 (6.1%) had a low, 473 (28.7%) a medium, and 1,072 (65.1%) a high educational background according to their self-reported highest educational degree. Sample characteristics for the three subgroups are detailed in Table S1.

Table S1. Baseline sample characteristics for participants with a low, medium, and high educational background

| | 9 or less years of school education | 10 to 11 years of school education | 12 or more years of school education |
|---|---|---|---|
| | (n=101) | (n=473) | (n=1,072) |
| | | | |
| **Women**, n (%) | 47 (46.5%) | 253 (53.5%) | 620 (57.8%) |
| **Age**, M (SD) | 29.0 (9.2) | 36.3 (12.1) | 28.9 (9.5) |
| **Employment status**, n (%) | | | |
| Full-time employed | 39 (38.6%) | 294 (62.2%) | 356 (33.2%) |
| Part-time employed | 11 (10.9%) | 86 (18.2%) | 261 (24.4%) |
| In education still | 8 (7.9%) | 26 (5.5%) | 410 (38.2%) |
| Unemployed | 18 (17.8%) | 23 (4.9%) | 12 (1.1%) |
| Other | 25 (24.8%) | 44 (9.3%) | 33 (3.1%) |
| **Cigarettes per day**, M (SD) | 11.4 (9.2) | 4.9 (7.5) | 1.4 (4.0) |
| **Alcohol risk level**, n (%) | | | |
| Low-risk alcohol use | 72 (71.3%) | 351 (74.2%) | 662 (61.8%) |
| At-risk alcohol use | 29 (28.7%) | 122 (25.8%) | 410 (38.2%) |
| **Drinks per week**, M (SD) | 2.3 (6.2) | 1.7 (3.5) | 2.4 (3.9) |
| **Study group**, n (%) | | | |
| Intervention group | 52 (51.5%) | 248 (52.4%) | 515 (48.0%) |
| Control group | 49 (48.5%) | 225 (47.6%) | 557 (52.0%) |

Participants with 12 or more years of school education who received the brief alcohol intervention (BAI) increased their weekly alcohol use from 2.3 alcoholic standard drinks (*SD* 3.6) at baseline to 2.7 drinks (*SD* 4.5) at month 12. BAI group participants with 10 to 11 years reported the same average number of alcoholic drinks per week at baseline (*M* 1.8, *SD* 3.5) and at month 12 (*M* 1.8, *SD* 3.2). Those with 9 or less years of school education who received the intervention reported an average of 1.8 drinks (*SD* 4.4) at

baseline and 2.7 drinks (*SD* 5.8) at month 12. Control group participants with 12 or more years of school education increased their weekly alcohol use from 2.4 drinks (*SD* 4.1) at baseline to 2.8 drinks (*SD* 5.6) at month 12. An increase was also observed in control group participants with 10 to 11 years of school education who reported an average of 1.6 drinks (*SD* 3.4) at baseline and 2.2 drinks (*SD* 4.2) at month 12. Those with 9 or less years of school education in the control group reported an average of 2.7 drinks (*SD* 7.7) at baseline and 2.4 drinks (*SD* 3.7) at month 12.

There was no intervention effect after 12 months in participants with 12 or more years of school education (*IRR* 0.95, *95% CI* 0.82-1.10; $BF_{H(0, 0.14)}$ 0.30). The findings for those with 10 to 11 ($BF_{H(0, 0.14)}$ 2.80) or 9 or less years of school education ($BF_{H(0, 0.14)}$ 1.40) indicated that the data were insensitive for intervention effects after 12 months. Although the null was always included in the confidence intervals (Table S2), the findings supported the notion that participants with 10 to 11 years of school education benefitted from the intervention (*IRR* 1.28, *95% CI* 0.97-1.68; *P*=0.076). The difference regarding the intervention effect after 12 months between those with 10 to 11 and those with 12 or more years of school education was marginally significant (*IRR* 1.35, *95% CI* 0.99-1.82; *P*=0.055). As there were only 101 participants with 9 or less years of school education in the trial, no reliable conclusions can be drawn from the data regarding BAI effects in this subgroup, as indicated by a Bayes Factor close to 1.

Table S2. Intervention effects over 12 months for participants with a low, medium, and high educational background[a]

| | Difference between intervention and control group[b] | | |
|---|---|---|---|
| | 9 or less years of school education | 10 to 11 years of school education | 12 or more years of school education |
| | | | |
| **Active intervention phase** | | | |
| Month 3 | 1.08 (0.53-2.19) | 1.08 (0.84-1.39) | 0.97 (0.84-1.12) |
| Month 6 | 2.17 (0.73-6.41) | 1.27 (0.97-1.69) | 1.08 (0.92-1.25) |
| **Follow-up** | | | |
| Month 12 | 1.60 (0.77-3.32) | 1.28 (0.97-1.68) | 0.95 (0.82-1.10) |

[a] Latent growth model (*N*=1,646) with higher-order growth factors for negative binomial distributed outcome data. The outcome was net changes in number of alcoholic drinks per week. The model was adjusted for sex, age, employment status, smoking, and alcohol-related risk level.

[b] Incidence Rate Ratios (IRRs) with 95% confidence intervals are displayed.
